# Experimental evidence for structured information–sharing networks reducing medical errors

**DOI:** 10.1073/pnas.2108290120

**Published:** 2023-07-24

**Authors:** Damon Centola, Joshua Becker, Jingwen Zhang, Jaya Aysola, Douglas Guilbeault, Elaine Khoong

**Affiliations:** ^a^Annenberg School for Communication, University of Pennsylvania, Philadelphia, PA 19104; ^b^School of Engineering and Applied Sciences, University of Pennsylvania, Philadelphia, PA 19104; ^c^Leonard Davis Institute of Health Economics, University of Pennsylvania, Philadelphia, PA 19104; ^d^Network Dynamics Group, University of Pennsylvania, Philadelphia, PA 19104; ^e^School of Management, University College London, London E14 5AA, United Kingdom; ^f^Department of Communication, University of California, Davis, CA 95616; ^g^Penn Medicine Center for Health Equity Advancement, University of Pennsylvania Health System and Perelman School of Medicine, University of Pennsylvania, Philadelphia, PA 19104; ^h^Haas School of Management, University of California, Berkeley, CA 94720; ^i^Center for Vulnerable Populations at San Francisco General Hospital, University of California, San Francisco, CA 94110; ^j^Division of General Internal Medicine at San Francisco General Hospital, University of California, San Francisco, CA 94110

**Keywords:** decision-making, collective intelligence, networks, medical errors

## Abstract

Errors in clinical decision-making are disturbingly common. Recent studies have found that 10 to 15% of all clinical decisions regarding diagnoses and treatment are inaccurate. Here, we experimentally study the ability of structured information–sharing networks among clinicians to improve clinicians’ diagnostic accuracy and treatment decisions. We use a pool of 2,941 practicing clinicians recruited from around the United States to conduct 84 independent group-level trials, ranging across seven different clinical vignettes for topics known to exhibit high rates of diagnostic or treatment error (e.g., acute cardiac events, geriatric care, low back pain, and diabetes-related cardiovascular illness prevention). We compare collective performance in structured information–sharing networks to collective performance in independent control groups, and find that networks significantly reduce clinical errors, and improve treatment recommendations, as compared to control groups of independent clinicians engaged in isolated reflection. Our results show that these improvements are not a result of simple regression to the group mean. Instead, we find that within structured information–sharing networks, the worst clinicians improved significantly while the best clinicians did not decrease in quality. These findings offer implications for the use of social network technologies to reduce errors among clinicians.

## Significance

Errors in clinical decision-making are disturbingly common. Here, we show that structured information–sharing networks among clinicians significantly reduce diagnostic errors, and improve treatment recommendations, as compared to groups of individual clinicians engaged in independent reflection. Our findings show that these improvements are not a result of simple regression to the group mean. Instead, we find that within structured information–sharing networks, the worst clinicians improved significantly while the best clinicians did not decrease in quality. These findings offer implications for the use of social network technologies to reduce diagnostic errors and improve treatment recommendations among clinicians.

Errors in clinicians’ diagnostic assessments are a leading cause of incorrect treatment recommendations (1, 2), resulting in both unnecessary testing and delayed treatment (3). The rapidly expanding literature on “medical collective intelligence” shows that the collective judgment of a large group of clinicians is consistently more accurate than judgments of expert individual clinicians (4, 5). This finding, sometimes referred to as “the wisdom of the clinical crowd”, has been demonstrated across numerous medical specialties, including dermatology (6), radiology (7), cardiology (8), and intensive care medicine (9). Growing interest in these findings among both academic and clinical researchers (10, 11) has motivated the development of new medical technologies and clinical protocols to harness the wisdom of the crowd for clinical decision-making (12–14).

However, a significant theoretical challenge for the expanding field of medical collective intelligence comes from the fact that research on large-scale crowd-based aggregation methods does not identify any mechanisms by which the “wisdom of the clinical crowd” improves individual clinical decisions. In other words, while aggregation methods can successfully demonstrate that a collective ‘group choice’ typically outperforms the individual members of the group, the group members themselves do not improve. This is partially a consequence of the fact that the vast majority of these studies are conducted post hoc (i.e., examining pools of data on clinicians’ past decisions); thus calculations of crowd wisdom are disconnected from the question of how crowd wisdom influences the quality of care offered by individual clinicians. This disconnect represents a significant problem for the field of medical collective intelligence. Without an approach for leveraging the increased medical intelligence at the ‘crowd level’ and a demonstration of the mechanisms through which it can advance the quality of individual clinical decisions, crowd research will have limited impact on real-time clinical reasoning.

We followed prior research in nonclinical settings by adopting a network-theoretic approach to studying how the aggregation dynamics of collective intelligence within large groups of clinicians may directly improve the quality of individual clinical judgements. Previous nonclinical studies have shown that structured information exchange networks with uniform—i.e., egalitarian (15)—connectivity can effectively translate group-level collective intelligence into real-time improvements in the quality of individual group members’ judgments (15–18). We explored whether this approach to leveraging the network aggregation dynamics of collective intelligence would directly improve the quality of individual clinical judgements. Historically, a significant limitation to testing this network-theoretic approach to medical collective intelligence was the impossibility of enrolling large groups of clinicians to participate in the evaluation of clinical cases simultaneously, while embedded in large information-sharing networks. However, recent advances in experimental network science have provided a method for connecting large numbers of doctors into information-sharing networks. This method was recently applied to the problem of reducing race and gender bias in clinical reasoning (19). The findings showed that networks produced significant reductions in medical bias as a result of clinicians’ interactions within egalitarian peer-to-peer information-sharing networks. Here, we use this network method across a range of clinical specialties to identify a general mechanism through which the network dynamics of medical collective intelligence can improve real-time medical judgements among large numbers of individual clinicians.

We recruited 2,941 practicing clinicians to participate in an online vignette–based experiment administered through a proprietary mobile networking app for clinicians (hereafter “App”; see *SI Appendix*, *Materials and Methods*). To evaluate the robustness of our experimental findings, we tested our network hypothesis across 7 different clinical cases, conducting a total of 84 replicated experimental trials (*SI Appendix*, *Materials and Methods*).

## Experimental Design

For each of the seven clinical case vignettes, we conducted a total of 12 trials—eight network trials and four control trials (*SI Appendix*, *Materials and Methods*). Each trial contained 40 clinicians. Each clinical vignette was reviewed by board-certified clinicians from three specialties: internal medicine, emergency medicine, and cardiology. Case vignettes were designed using clinical decision-making scenarios which were known to elicit errors despite having an accepted, evidence-based risk estimate and treatment recommendation. In six out of seven vignettes, the correct estimate and treatment recommendation were identified by existing society guidelines for evidence-based care. In the case that no society guidelines existed (i.e., vignette three), the correct responses were identified by available evidence-based research (see *SI Appendix*, *Clinical Vignettes* for details). The clinical case scenarios used in this study included: acute cardiac events, geriatric care and decline in activities of daily living, lower back pain, and diabetes-related cardiovascular illness prevention (*SI Appendix*, *Materials and Methods*).

Our experimental approach to studying collective intelligence in real-time medical decision-making is based on past work that identifies an essential connection between risk estimation processes and correct treatment decisions in clinical reasoning (20). This work shows that a clinician’s categorical treatment decision, for instance to either admit a patient to the observation unit for further testing, or to send them home with a 1-wk follow-up, relies upon an (often unconscious) estimate of a patient’s likelihood of suffering an adverse health event within a clinically relevant time window (20). This model of medical reasoning indicates that although a clinician must ultimately make a categorical choice by selecting a single treatment plan, the underlying rationale for their decision is typically based upon a risk estimate for the patient. An important implication of this model is that the quality of clinicians’ treatment decisions may be directly improved by increasing the accuracy of their clinical estimates about a patient’s risk level (19, 20). We use this insight in the development of our experimental networking protocol for clinicians by requiring participating clinicians to provide, for each patient: i) an estimate of the patient’s risk level (i.e., a diagnostic assessment of a patient’s likelihood of having an adverse health event within a stipulated time period, from 0 to 100), and ii) a categorical treatment decision (i.e., a recommendation for a treatment plan for the patient). This experimental design enables us to identify the process through which medical collective intelligence may produce direct changes in individual clinicians’ medical judgments both in terms of the accuracy of their estimates regarding patients’ risk levels, and the quality of their ultimate treatment recommendations.

Participating clinicians in the study viewed the case vignettes using the study App, and were instructed to respond to two separate questions: one pertaining to diagnostic risk estimate and one pertaining to treatment recommendation. For instance, for a clinical case concerning cardiac illness, the diagnostic question asks, “What is the estimated 6-wk risk of this patient having a major adverse cardiac event?” The treatment decision question then asks, “What is your recommended next step: A) discharge, B) admit for evaluation or C) admit for observation unit.”

**Fig. 1.**
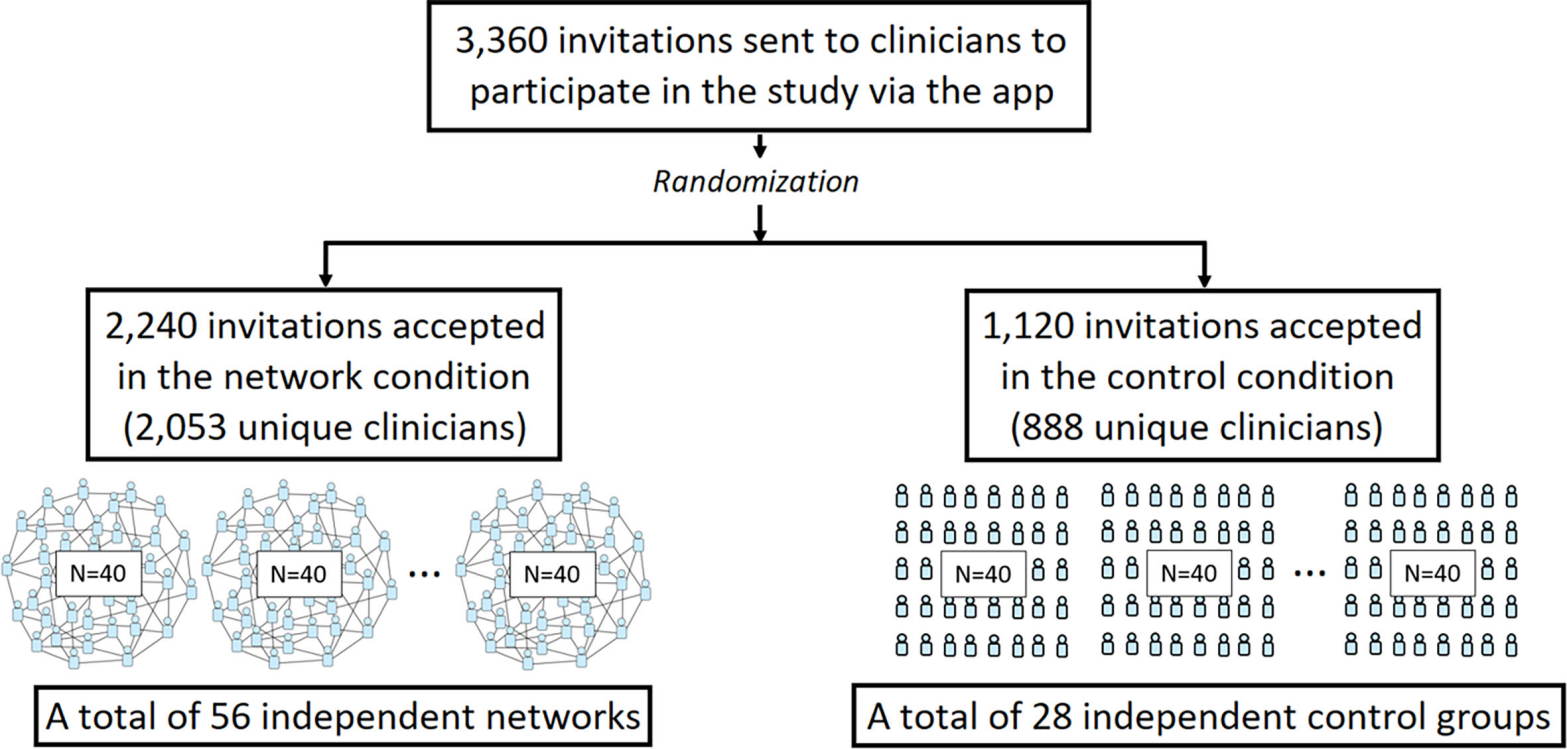
Participant flow through the study.

In each trial, participants were digitally randomized to either the network condition or the independent control condition in a 2:1 ratio (Fig. 1). In the network condition, participants were randomly assigned to a single location in a large, anonymous egalitarian online peer-to-peer network (*N =* 40 clinicians per network), in which every participant had an equal number of connections (*z* = 4 connections per clinician), which ensured that no single participant had greater power over the communication dynamics within the network (18) (*SI Appendix*, *Materials and Methods*). Participants in the network condition were told that they would see clinical estimates from anonymous peers. Following previous studies of network learning dynamics (16, 18, 19), we did not provide participants with information about the size of their immediate network neighborhood, nor the overall size of the network in which they were embedded. Within each trial, participant networks were fixed; connections did not change during the course of a trial. In the control condition, clinicians (*N* = 40 per trial) viewed the same vignette and answered the same questions as in the network condition, but they acted independently, and were not embedded in social networks.

In both the network and control conditions, participants were given three rounds to provide their risk assessments and treatment recommendations for the presented clinical case. In the initial round, all participants independently viewed the case vignette, and were given 2 min to provide their risk assessment and treatment recommendation. In the network condition, in round two clinicians were again shown the case vignette, as well as being shown the average risk estimate given by their network contacts. They were then asked to provide a revised risk estimate and clinical recommendation. Notably, participants were only provided with information about their peers’ risk assessments, and were not given information about their peers’ treatment recommendations. Per earlier research on medical decision-making (20), this experimental design enabled us to evaluate whether information-sharing networks in which clinicians share only their risk estimates can yield direct improvements in the quality of their treatment recommendations. In the second round, participants could either provide the same responses they gave in the initial round or modify their responses. In the final round, this procedure was repeated again. Participants were shown the average risk estimate given by their network contacts in the second round, and were then asked to provide their final risk estimates and treatment recommendations.

The control condition was identical to the network condition, except that clinicians were not embedded in peer networks, and were not given any information about other participants’ responses. Thus, in the control condition, in all three rounds participants remained isolated while they viewed the clinical vignette, and provided their risk estimate and treatment recommendation. In all three rounds, participants were given 2 min to respond.

Each trial lasted approximately 8 min. All participants were eligible to participate in up to seven trials (one trial for each of the seven clinical cases). The vast majority of subjects (70%) participated in only one trial (i.e., one clinical case). A minority of subjects (30%) participated in two independent trials. These subjects were independently randomized to condition in each trial. No subjects participated in more than two trials. Upon completing a trial, participants were shown a final payment page that displayed: 1) the amount of compensation, ranging from $0 to $40, based on the accuracy of their response in the final round; 2) the correct treatment recommendation for the patient; and 3) resources for learning more about the case examined in the clinical vignette (*SI Appendix*, *Materials and Methods*). All participants, regardless of experimental condition and compensation, were provided with the same final information and resources concerning the correct answers and *SI Appendix*, *Materials and Methods* for each vignette (*SI Appendix*, *Materials and Methods*).

## Analysis Approach

Our primary outcomes of interest are changes in the accuracy of clinicians’ risk estimates, and changes in the overall frequency of clinicians making the correct treatment recommendations. First, we determined the error of each clinician’s estimate at each round by calculating the absolute distance (in percentage points) between their provided estimate and the correct estimate for the patient vignette. We converted this measure of diagnostic error into a measure of diagnostic accuracy by i) taking the negative of each clinician’s diagnostic error (ε_i_ × −1 for all clinicians *i*, …, *n*), so that clinicians with the highest error (i.e., the greatest absolute distance between their estimate and the truth) are considered to have the lowest accuracy, and then ii) arranging these negative error values along a 0 to 1 scale using min–max normalization [*x* – min(*x*)/max(*x*) – min(*x*)]. Finally, we adopted the conservative strategy of calculating diagnostic accuracy at the trial level. This conservative analytic approach reduces our sample size, and thus reduces our power to detect the effects of our network intervention on clinician performance; however, it is the most rigorous way to account for the nonindependence among clinicians within the network condition (*SI Appendix*, *Materials and Methods*), and therefore enables us to preserve causal identification of the direct effects of egalitarian peer networks on changes in clinician performance (15, 16, 18, 19). Second, we report the fraction of clinicians in each trial providing the correct clinical recommendation. As above, to preserve causal identification of the direct effects of networks on changes in clinical performance, these results are also evaluated at the trial level, measured as the percentage of clinicians making the correct recommendations in their final responses per condition in each trial.

We conducted 84 independent trials of this study. All statistical analyses were conducted at the trial level. Our randomization scheme produced 56 trial-level observations in the network condition, and 28 trial-level observations in the control condition (additional details in *SI Appendix*, *Materials and Methods*). Power calculations determined that 56 trials (each with 40 clinicians) in the network condition, and 28 trials (each with 40 clinicians) in the control condition, would have greater than 90% power to detect the anticipated effect size (Cohen’s *d* = 0.207) based on prior studies (15, 16, 18). We used the Wilcoxon rank sum test to compare the network and control condition; and we used the Wilcoxon signed-rank test to compare changes within each trial of each condition, paired at the trial level. The trial-level analyses controlled for statistical nonindependence among clinicians in the social network condition (*SI Appendix*, *Materials and Methods*) (15–18).

Fig. 1 shows the participant flow of this study. A total of 3,360 invitations were sent through push notifications to clinicians to join the study via the App. Once clinicians clicked on the push notification, they were randomized to either the network or control condition via the App. Then, 2,240 clinicians were randomized to the network condition, and 1,120 clinicians were randomized to the control condition. After accounting for attrition and for clinicians who participated in multiple trials, 2,053 unique clinicians in total joined and completed the task in the network condition, and 888 unique clinicians in total joined and completed the task in the control condition. Only 30% of clinicians participated in multiple clinical cases, and clinicians participated in at most two of the seven clinical trials (see *SI Appendix*, *Materials and Methods* and Fig. S1 for randomization details on the intention-to-treat sample). There were no significant differences in clinician characteristics across experimental conditions (Table 1).

**Table 1.** Participating clinicians’ characteristics across the two experiment conditions

	Experiment Condition: n (%)	
	Network condition 2053 Unique clinicians	Independent Control 888 Unique clinicians
**Gender**		
Male	63.2%	74.1%
Female	36.7%	25.8%
**Date of NPI Assignment**		
2017	54.7%	52.2%
2013–2016	19.6%	23.8%
2009–2012	14.0%	15.0%
2005–2008	11.5%	8.8%
**Primary Care**	89.3%	84.3%
**Independent Practice**	25.4%	16.9%

## Results

Our findings show that the accuracy of diagnostic assessments significantly improved in both conditions. Over all trials, at baseline, the average estimate accuracy was 76.8% (error of 23.2% points) in the independent control condition, and 76.3% in the network condition (error of 23.7% points), exhibiting no significant differences across conditions (*P* = 0.9, Wilcoxon rank sum test). By the final round, average accuracy increased to 79.3% (error of 20.9% points) in the control condition (+2.5% point improvement, *P* < 0.001), and 81.3% in the network condition (+5.0% point improvement, *P* < 0.001) (Wilcoxon signed-rank test, two-tailed). While the opportunity to revise based on independent reflection led to a significant increase in the diagnostic accuracy in clinicians’ assessments, the network condition produced significantly greater increase in diagnostic accuracy, doubling the percentage point improvement compared to the control condition (+2.5% point improvement, *P* < 0.01, Wilcoxon rank sum test, two-tailed).

**Fig. 2.**
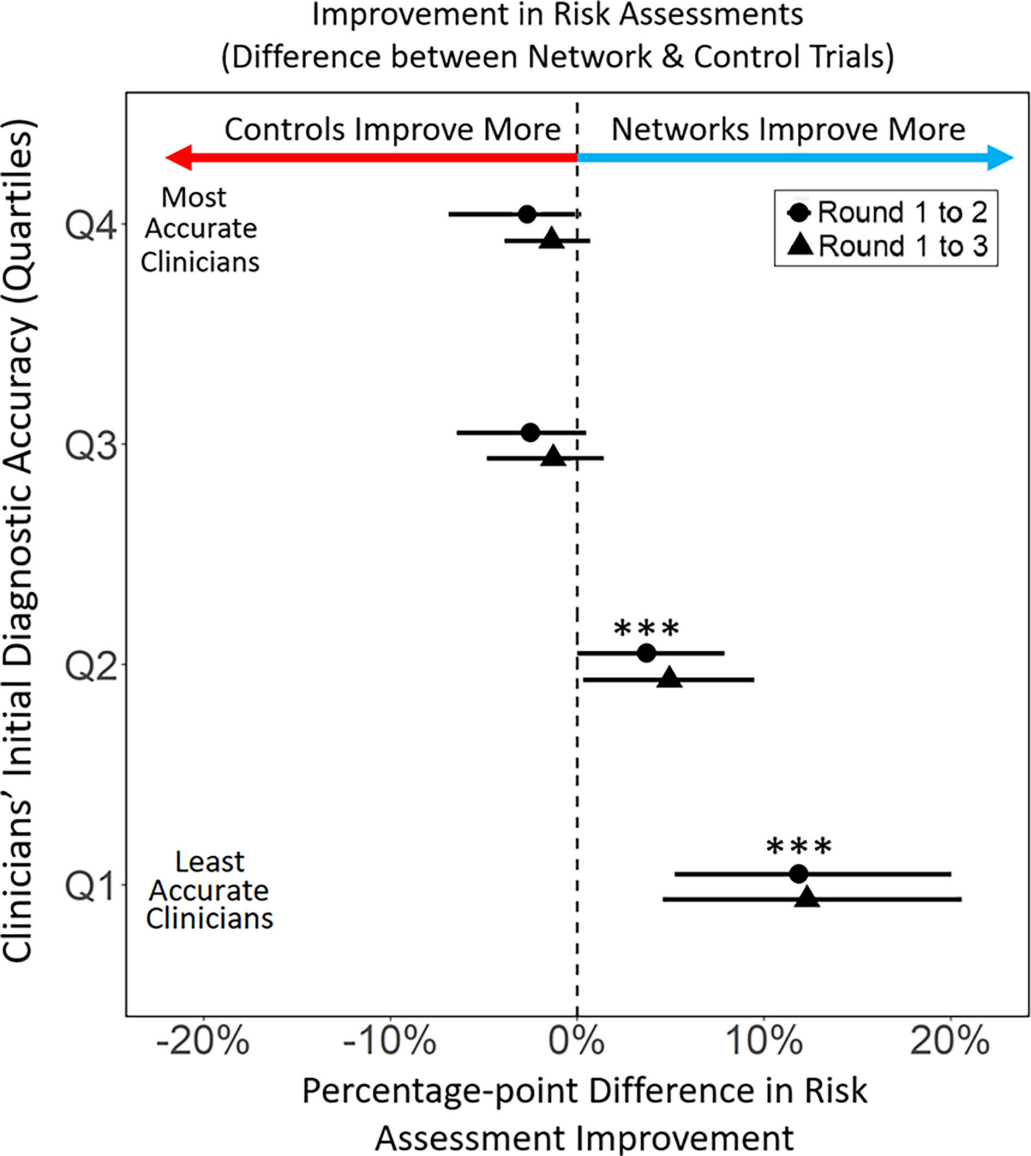
Differential improvement in diagnostic assessments comparing the network condition and the control condition. Differential effects of experimental conditions on changes in the accuracy of clinicians’ diagnostic assessment; circles represent the change from round 1 to 2, and triangles represent the total change from round 1 to 3. Clinicians are grouped into quartiles based on the accuracy of their initial (round 1) diagnostic assessments, ranging from least accurate (Q1) to most accurate (Q4). Decentralized information–sharing networks had the greatest effect on increasing accuracy of assessments among the initially least accurate clinicians. Change in accuracy is displayed as percentage points, where accuracy is represented from 0 to 100% using min–max normalization. Error bars represent 95% CI. Differences between conditions within each quartile, along with confidence intervals, are estimated using the Wilcoxon rank-sum test.

Of paramount interest is how improvements in accuracy within structured information–sharing networks varied according to the quality of physicians’ initial responses. Were improvements primarily among the best performing clinicians, or did structured information sharing serve to improve the performance of the initially least accurate clinicians? Most importantly, did improvements by some come at the cost of significant reductions in the performance of others (e.g., as would emerge in an averaging process that increased the accuracy of the poorest performers, but reduced the accuracy of the best performers)? Fig. 2 shows that this did not happen. Overall changes in the accuracy of clinicians’ responses are shown from round 1 (initial response) to round 3 (final response), for all clinicians according to the error of their initial responses, which are broken down by quartiles (SI). Interestingly, the results show that networks did not produce a simple averaging effect. The most accurate clinicians, who were initially in the top quartile of diagnostic assessment accuracy (Q4) exhibited no significant effects of information-sharing networks—showing no significant change in accuracy across experimental conditions. Similarly, clinicians in the third quartile of accuracy (Q3) also showed no significant change in diagnostic accuracy (*p* = 0.39, Wilcoxon signed-rank test) across experimental conditions. However, in the lower half of the group, among clinicians in the second quartile (Q2) lower performing clinicians in information-sharing networks significantly increased accuracy compared to clinicians in the control condition (+4.04% point increase, *P* < 0.04, Wilcoxon signed rank test, two-tailed). Similarly, the least accurate clinicians (Q1) showed the greatest differential between the control and the network condition. Diagnostic accuracy increased +14.5% points more in the network condition than in the control condition (*P* < 0.001, Wilcoxon signed rank test, two-tailed). These results indicate that while the most accurate clinicians (Q3 and Q4) were not adversely affected by social influence, diagnostic accuracy among the least accurate clinicians (Q1 and Q2) significantly increased as a result of information-sharing networks.

**Fig. 3.**
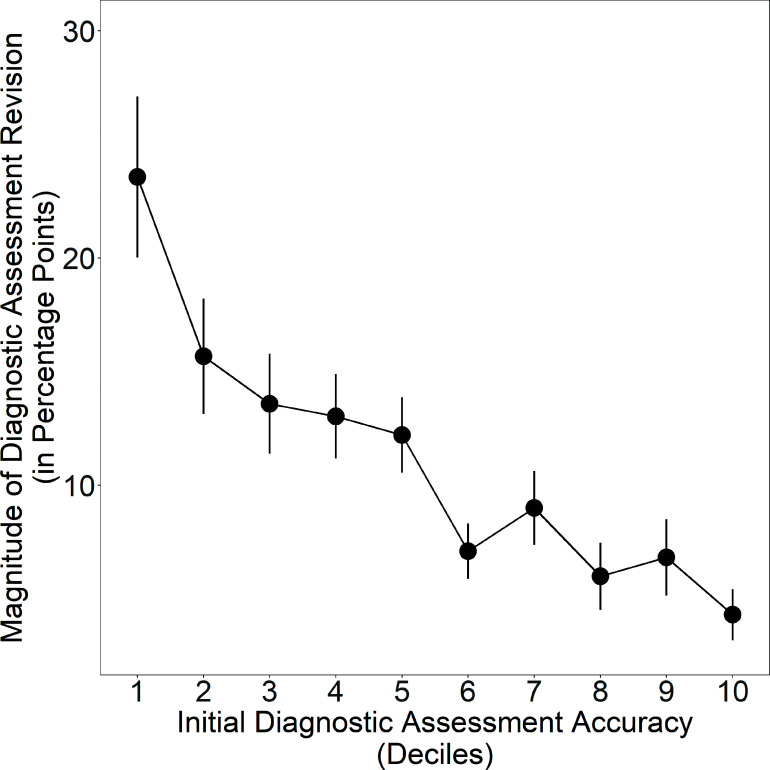
Clinicians’ propensity to revise their diagnostic assessments in the network condition according to their initial diagnostic accuracy, binned by deciles (1 is least accurate, 10 is most accurate). Clinicians’ accuracy in their initial assessment significantly predicts the magnitude of their revisions to their diagnostic assessments from their initial to final response. Error bars display 95% CI.

The network mechanism that explains the improvements in clinicians’ diagnostic assessments is the disproportionate impact of accurate individuals in the process of belief revision within egalitarian social networks (15–18). As demonstrated in earlier studies of networked collective intelligence (17, 18), during the process of belief revision in decentralized information-sharing networks, there is an expected correlation between the accuracy of an individual’s beliefs and the magnitude of their belief revisions, such that accurate individuals revise their responses less; this correlation between accuracy and revision magnitude is referred to as the “revision coefficient” (18). Within decentralized social networks, a positive revision coefficient has been found to give greater de facto social influence to more accurate individuals, which is predicted to produce network-wide improvements in the accuracy of individual beliefs within egalitarian social networks. In past studies, these improvements in collective accuracy have been found to result in a corresponding reduction in errors among initially inaccurate participants (16–18). Fig. 3 tests this prediction for clinicians in our study. The results show, as expected, that there is a significant positive revision coefficient among clinicians in the network condition (*P* < 0.001, Jonckheere–Terpstra test, two-tailed), indicating that less accurate clinicians made greater revisions to their responses while more accurate clinicians made smaller revisions, giving greater de facto influence in the social network to more accurate clinicians (See *SI Appendix*, Fig. S7, for additional details). All of these results are summarized in Table 2 (see *SI Appendix*, Table S1 for additional breakdown by vignette).

**Table 2.** Summary statistics for outcome measures by initial assessment error quartiles and by experiment conditions, averaged across trials at the individual level (see SI Appendix, Table S2 for trial level comparisons)

Experiment Condition	Initial Error Quartile	Initial Assessment Accuracy	Final Assessment Accuracy	Change	Initial Rate in Correct Treatment	Final Rate in Correct Treatment	Change
Control	Q1 (error)	97.2	94.6	−2.60	0.80	0.81	+0.01
Control	Q2 (error)	88.9	89.5	+0.05	0.45	0.51	+0.06
Control	Q3 (error)	71.1	74.6	+3.50	0.34	0.38	+0.04
Control	Q4 (error)	47.2	55.9	+8.70	0.25	0.32	+0.06
Network	Q1 (error)	97.1	93.9	−3.16	0.76	0.78	+0.02
Network	Q2 (error)	89.0	87.8	−1.22	0.44	0.48	+0.04
Network	Q3 (error)	72.2	79.0	+6.84	0.33	0.43	+0.10
Network	Q4 (error)	46.1	62.9	+16.9	0.23	0.35	+0.13

Notes: Q4 (error) denotes the group of clinicians who are in the highest quartile of error (bottom quartile of accuracy) for initial diagnostic assessment. Q2 (error), Q3 (error), and Q4 (error) denote the second, the third, and the highest quartile of error, respectively.

**Fig. 4.**
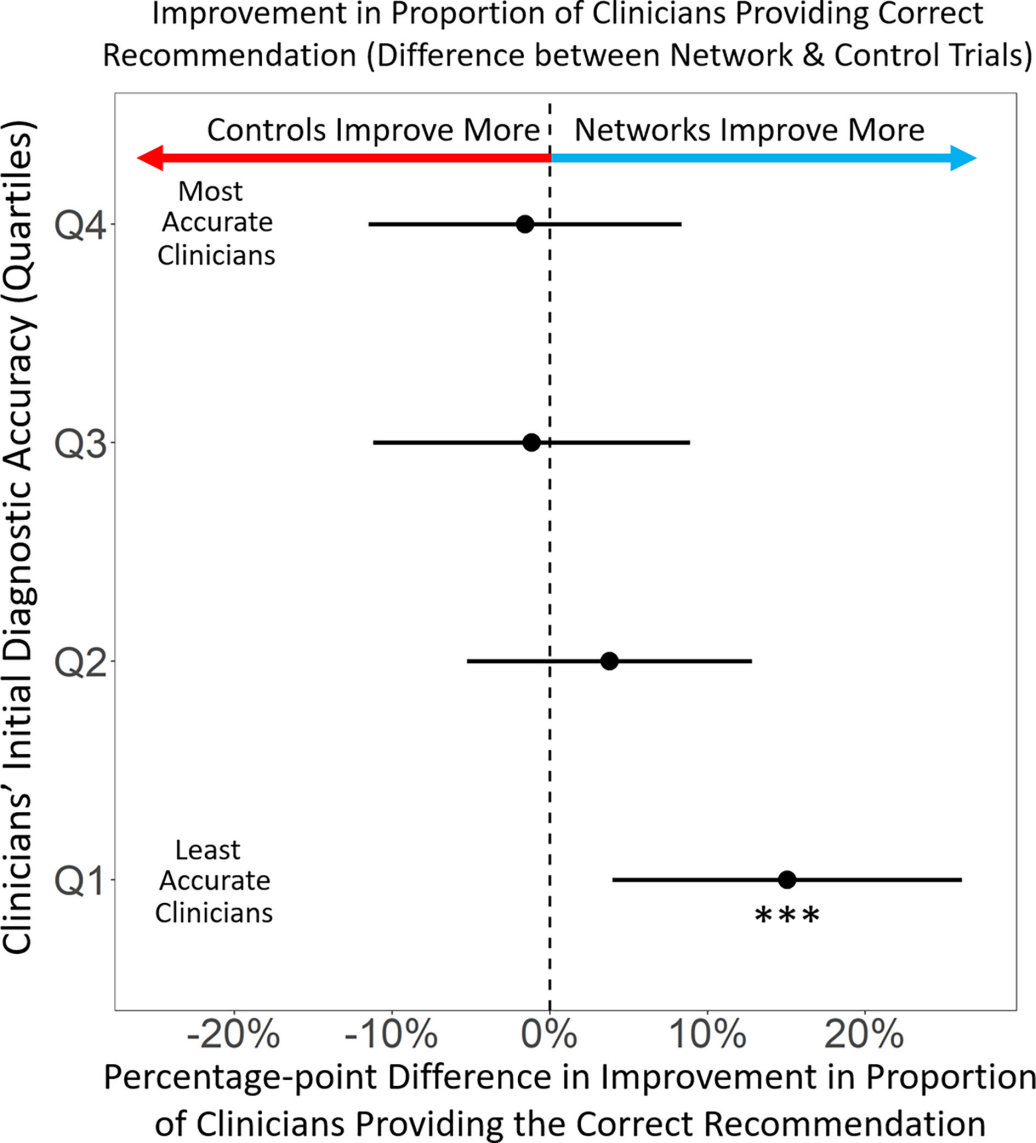
Differential improvement in correct treatment recommendations comparing the network condition and the control condition. Differential effects of experimental conditions on changes in the proportion of clinicians providing the correct clinical recommendation, from round one to round three. Clinicians are grouped into quartiles based on the accuracy of their initial (round 1) diagnostic assessments, ranging from least accurate (Q1) to most accurate (Q4). Decentralized information–sharing networks had a significant effect on improving correct clinical recommendations among the initially least accurate clinicians. Error bars represent 95% CI.

As predicted, improvements in assessment accuracy among clinicians translated directly into improvements in clinical treatment recommendations, resulting in an increased likelihood of clinicians switching from an initially incorrect treatment recommendation to the correct recommendation in their final response (*P* < 0.001, *r_s_* = 0.25). To examine the effects of information-sharing networks on the correctness of clinicians’ final treatment recommendations, we evaluated the change in the proportion of clinicians providing the correct answer at round one versus round three. Overall, we found significant increases in the proportion of clinicians providing the correct clinical recommendation, in both the control (by +4.3% points, *P* = 0.02, *n =* 28) and the network condition (+7.3% points, *P* < 0.0001, *n =* 56) (Wilcoxon signed rank test, two-tailed). Fig. 4 shows that for clinicians in the top 3 quartiles (Q2, Q3, and Q4) there were no significant differences between the control and the network condition in the proportion of clinicians changing their recommendations from an incorrect recommendation to the correct treatment (*P* = 0.75 for Q4; *P* = 0.82 for Q3; *P* = 0.4 for Q2). However, among the least accurate clinicians (Q1), there was a large and significant (+15% point, *P* < 0.01, Student *t* test, two-tailed) increase from the control condition to the network condition in the fraction of clinicians who changed their responses from an initially incorrect treatment recommendation to the correct treatment recommendation in their final responses. These findings indicate that structured peer network influences on clinicians’ diagnostic risk assessments significantly improved the quality of clinicians’ treatment recommendations, particularly among poor performing clinicians.

## Discussion

Our study differs from previous studies of collective medical decision-making. Instead of using simulated retrospective analyses to aggregate clinical decisions into a wisdom of the crowd calculation (4, 6, 21–23), we demonstrate how the network dynamics of medical collective intelligence may directly improve clinicians’ individual and collective clinical decision-making. Our control condition showed results consistent with prior literature on collective intelligence, in which the average performance of an aggregated group of independent clinicians outperformed individual clinicians (sometimes referred to as the “wisdom of the crowd”) (18, 19, 24–27). Nevertheless, we found that the clinicians in the control condition were consistently outperformed by clinicians in the network condition, indicating that the network dynamics of social learning in structured egalitarian networks improved the medical collective intelligence found among groups of independent clinicians (16, 18). Within the network condition, improvements in clinical decision-making were greatest among clinicians with the greatest initial error, while the best performing clinicians retained their initial high-quality decision-making. Thus, we did not find a regression to the mean (18), in which the network dynamics that improved decision-making by the worst-performing clinicians had a symmetrical “lowering” influence on the decision-making from the best-performing clinicians. An interesting direction for future research based on our findings is to explore the degree to which network learning among clinicians may engage different cognitive processes than those used by clinicians in the control condition, such as “fast” vs. “slow” thinking (i.e., system 1 vs. system 2) (28). Further directions for future research include exploring the effects of different interaction technologies, such as chat tools, videoconferencing, etc., on the quality of clinical reasoning, as well as the interaction effects of providing participants with varying information about the size of their network (21), and the resulting impact of these changes on improving versus reducing clinicians’ collective intelligence.

As with all experiments, the design of our study enabled a careful identification of causal factors by limiting the behaviors that could be tested. We used case vignettes and therefore did not assess clinicians’ responses to actual patients. However, utilizing case vignettes is standard practice to assess clinician competency in clinical decision-making, such as during medical board certification exams (29, 30). This methodological choice is particularly appropriate because our goal was to assess changes in the quality of clinicians’ real-time reasoning consistent with standard approaches of evaluating medical decision-making (31–33). Our design also enforced an egalitarian architecture for the information-sharing networks in our study. We used this architecture because it has been found in previous studies to improve the efficiency of social learning (15, 18). However, we acknowledge that this egalitarian network architecture differs significantly from the information channels typically found in uncontrolled clinical settings, in which clinicians’ information-sharing networks often become highly centralized based on factors such as seniority, specialty and ranking in the medical community (34, 35). Previous studies have shown that while domain experts can increase group performance in some cases (11, 18, 36), increased network centrality can amplify errors in individual judgment, leading to significantly impaired social learning and reductions in collective intelligence (11, 17, 18). These results suggest that there may be unintended consequences of centralized information channels within uncontrolled medical contexts, which may possibly contribute to increases in diagnostic errors and mistreatment in those settings (11, 37). An important feature of this study was the ability to control the design of the information-sharing networks among clinicians to identify the direct effects of egalitarian peer information-sharing networks on clinician performance. One implication of our findings is that approaches to implementing medical collective intelligence through information-sharing networks in clinical settings may need to give close attention to the structure of the information-sharing architecture that is used.

Although we did not focus on implementation within this study, we anticipate that our findings offer several important opportunities for integrating medical collective intelligence into clinical decision-making procedures. Diagnostic errors are increasingly recognized as a critical source of error in healthcare, and were highlighted in a 2015 NASEM report on improving diagnosis (3). This report produced eight high-level recommendations to reduce diagnostic errors, two of which focused on the need for more effective collaboration and use of health information technologies to support the diagnostic process. Our findings provide evidence that effective collaboration channels for reducing diagnostic errors may be achieved through harnessing medical collective intelligence in real-time, egalitarian information-sharing networks. However, unlike the information-sharing channels that naturally occur in clinical communities, which are often based on consultations with senior clinicians, our study provides specific guidance on how to design a structured network architecture for health information technologies that is adapted to support improvements in diagnosis and treatment decision-making.

From a digital infrastructure perspective, the growing digitization of healthcare has resulted in a healthcare information technology infrastructure that could be adapted to include collaboration networks identical to the ones created for this study (38–40). Importantly, clinician acceptance of an intervention like this is greatly facilitated by the fact that many clinicians are accustomed to submitting and reviewing clinical cases on digital platforms (41–48). Digital case platforms are used not only for training purposes but also within current clinical care in the form of electronic consultations, or e-consults (49, 50).

E-consult technologies have been increasingly adopted within underresourced health systems, especially those with limited access to specialties (49–51). Within e-consult platforms, clinicians describe a clinical case and then submit it electronically for consultation with a specialist. Within a short timeframe (typically 24 to 48 h), the specialist then replies with their recommendations, and the referring clinician can then consider these recommendations when providing care for the patient. As more health systems adopt e-consults, we anticipate it will be feasible to harness medical collective intelligence from decentralized information-sharing networks within e-consult platforms. We anticipate, for instance, that instead of sending clinical cases to a single specialist, clinicians may instead submit cases to a network of specialists who participate in a structured information exchange process before providing a recommendation to the referring clinician. The clinician could then use the network feedback to inform their decision-making, consistent with current e-consults practices. Our findings indicate that a proper implementation infrastructure, as described in our study, integrated within existing healthcare technologies may enable referring clinicians from any geographic location to benefit directly from the striking improvements in patient care that come from real-time engagement with medical collective intelligence, yet without requiring changes to the current e-consults experience or timeline.

## Conclusion

Our study expands the growing field of research on medical collective intelligence by identifying the direct implications of leveraging network dynamics of collective learning within egalitarian networks for improving individual clinician decision-making in real-time. Our findings have the potential to improve clinical decision-making procedures for both trainees and practicing clinicians. Given the growth of health information technologies in recent years, we expect future studies to expand on our findings by exploring how various specialties in clinical medicine may benefit from this approach, and how best to develop applications within the rapidly expanding space of healthcare infrastructure and information technology.

## Materials and Methods

Participants were randomly assigned to one of two conditions: i) the “control condition” where clinicians provided diagnostic assessment estimates on their own, without any exposure to the estimates of other clinicians, or ii) the “network” condition, where clinicians were shown the average diagnostic estimates of other clinicians in a structured social media network. Each condition in each trial contained 40 clinicians. We conducted 56 independently replicated trials in the network condition, and 28 independently replicated trials in the control condition. If placed into a network condition, participants were randomly assigned to one node in a single network, and they maintained this position throughout the experiment. The network condition used a random network topology of 40 nodes with four edges per node. The same network topology was used across all trials in the network condition.

In each trial of each condition, clinicians were presented with a patient vignette and were asked to make an assessment about the medical condition of a patient by providing a probability estimate from 0 to 100 (*SI Appendix*, *Stimuli Design and Clinical Vignettes*). After providing a probability estimate, clinicians selected a treatment option from a dropdown menu specifying different courses of action. In the network condition, clinicians were not shown the treatment decisions made by other clinicians as a social signal; only the average probability estimate of each participant’s network neighbors was shown.

Upon registration, clinicians were encouraged to try five diagnostic challenges. Clinicians were able to try more than five challenges at their discretion by responding to push notifications when they were invited, but no clinician was invited to try the same vignette more than once. Each time clinicians arrived at a challenge, they were randomized between the control and the network condition. Because our statistical tests are based on between-participant comparisons and because participants were always randomized to conditions, our results are robust to repeated participants across trials.

This study was approved by the University of Pennsylvania’s Institutional Review Board, where the data collection for this study was based. All participants provided digital informed consent through the app before participating.

## Supplementary Material

Appendix 01 (PDF)

Dataset S01 (CSV)

Dataset S02 (CSV)

Code S01 (TXT)

## Data Availability

All data and code associated with this study can be downloaded at the following GitHub, https://github.com/drguilbe/CIdiagnosis (52). Anonymized spreadsheet with anonymized participant responses per round data have been deposited in https://github.com/drguilbe/ (53). All study data are included in the article and/or supporting information.
