# Supplementary material for: Experimental evidence for structured information–sharing networks reducing medical errors
Source: Proc Natl Acad Sci U S A. 2023 Jul 24;120(31):e2108290120. doi: 10.1073/pnas.2108290120 (PMC10401006; doi:10.1073/pnas.2108290120)
Supplement: Supplementary file 1 — Appendix 01 (PDF) [file pnas.2108290120.sapp.pdf]

Supplementary Materials for

Experimental Evidence for Structured Information-Sharing Networks Reducing Medical Errors

This PDF file includes:

- **Supplementary Materials and Methods**
- **Supplementary Figures**

Supplementary Materials and Methods

Experimental Design

Participants were randomly assigned to one of two conditions: (*i*) the "control condition" where clinicians provided diagnostic assessment estimates on their own, without any exposure to the estimates of other clinicians, or (*ii*) the "network" condition, where clinicians were shown the average diagnostic estimates of other clinicians in a structured social media network. Each condition in each trial contained 40 clinicians. We conducted 56 independently replicated trials in the network condition, and 28 independently replicated trials in the control condition. Power tests showed that fewer replications of the independent control groups were needed for the statistical comparison between the network condition and the control condition (1-3). If placed into a network condition, participants were randomly assigned to one node in a single network, and they maintained this position throughout the experiment. The network condition used a random network topology of 40 nodes with 4 edges per node. Past studies have shown that the findings from this topological structure are robust to variations in network size, network clustering and network density (i.e., average degree) (1-3). The same network topology was used across all trials in the network condition.

In each trial of each condition, clinicians were presented with a patient vignette and were asked to make an assessment about the medical condition of a patient by providing a probability estimate from 0–100 (see *Stimuli Design* and *Clinical Vignettes*, below). After providing a probability estimate, clinicians selected a treatment option from a dropdown menu specifying different courses of action. In the network condition, clinicians were not shown the treatment decisions made by other clinicians as a social signal; only the average probability estimate of each participant's network neighbors was shown.

Upon registration, clinicians were encouraged to play five diagnostic challenges. Clinicians were able to play more than five challenges at their discretion by responding to push notifications when they were invited, but no clinician was invited to play the same vignette more than once. Each time clinicians arrived at a challenge, they were randomized between the control and the network condition. Because our statistical tests are based on between-participant comparisons and because participants were always randomized to conditions, our results are robust to repeated participants across trials.

Recruitment

In total, 3360 participating clinicians were required by our study design. Clinicians were recruited from around the US by posting recruitment messages over clinician discussion boards on Reddit and paid targeted advertisements on Facebook. Seven recruitment messages were posted on Reddit, specifically on messaging boards that attract doctors and resident clinicians. Clinicians were also recruited through Penn Medicine's Graduate Medical Education training program (for resident clinicians). Advertisements were circulated to the 2017 cohort of resident clinicians, and clinicians were also recruited through outreach events as part of Penn Medicine's orientation for incoming residents. We also distributed three advertisements over Facebook, from December 10th 2017 to February 27th 2018, while making use of Facebook's advertising software to target clinicians. We limited advertisement exposure to people who resided in the US, who were 18 to 65, and whose demographic characteristics included the following features suggested by Facebook: healthcare and medical services, doctor (Dr), medical doctor (MD), medical doctor MD/medical director, and clinician. Since our study design allowed for clinicians to partake in multiple trials, each time undergoing independent randomization, fewer unique clinicians than the

overall sample were required to complete all trials. Just over 10% of clinicians failed to successfully join the task after responding to our push notification. In total, 2,941 unique clinicians participated in our study. Once in the task, 5.1% of participating clinicians dropped out of the task before it was finished. All of our main results continue to hold with equal statistical significance if we restrict our analyses only to those participating clinicians who completed the entire task in both the control and network condition.

Each advertisement directed clinicians to a webpage that specified the purpose of the research, eligibility requirement, and research compensation to interested participants (Figure S1). The webpage provided links to Google Play or the Apple app store, where participants could enroll by downloading the proprietary app called "DxChallenge" for free. The webpage informed clinicians that each diagnostic challenge would be announced via push notifications on their phone, which would appear on their screen and could be clicked to take them into the trial.

The original recruitment advertisement (Figure S1) indicated that clinicians could participate in up to 5 (of the total 7) trials. This limitation on the number of trials that clinicians could participate in was originally designed to manage overall project costs (limiting payouts to a maximum of $200 per participant: maximum of $40 per trial x 5 trials). However, we found upon commencing the study that clinicians rarely participated in more than one trial, and thus we allowed participants to enroll in any of the 7 trials since the 5 trial restriction was superfluous.

The typical time for participants to complete each trial was 5 minutes (when all members of a trial finished each round in under 2 minutes), however during the study we found that some trials lasted up to 8 minutes. We report 8 minutes in the main text since this is the maximum amount of time used for any trial.

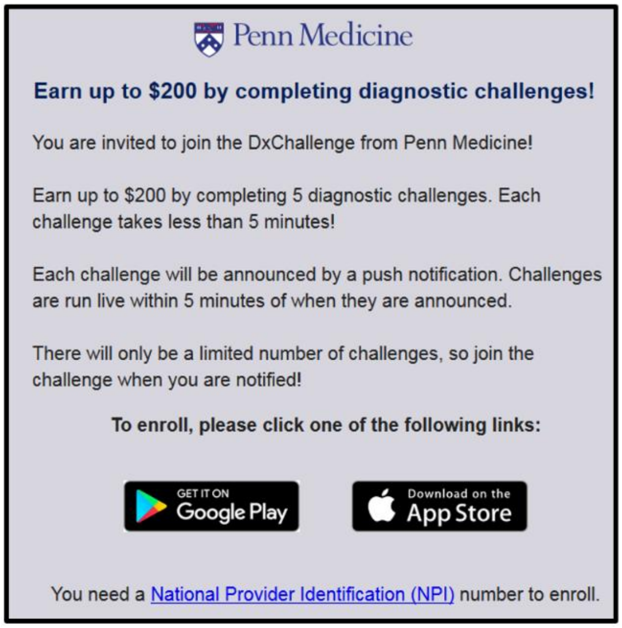

Figure S1. Displayed is a screenshot of the webpage where participants learned about the DxChallenge app and were provided with links to app stores where they could download the app for free.

When registering in the app, participants were required to input a valid email address and a valid 10-digit National Provider Identification (NPI), i.e., the unique personal identifier given to health care providers in the US. Each NPI could be queried in a public registry to obtain the state in which a given clinician was registered to practice as a health care provider. Using this information, we were able to determine that the NPI's for clinicians in our study pool originated from 46 states (Figure S2).

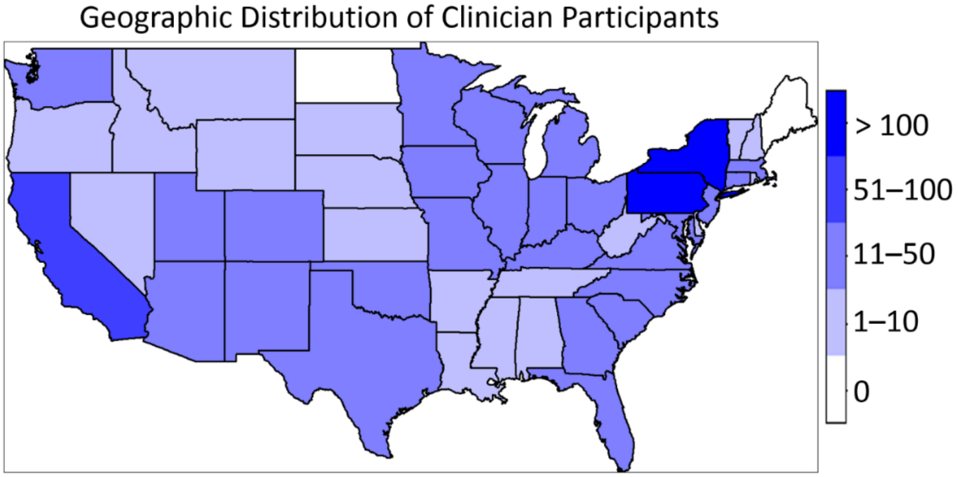

Figure S2. Displayed is the geographic distribution of clinicians from across the US who registered to participate in this study using the DxChallenge app. The geographic distribution of our participant pool was determined using the NPI ('National Provider Number') of each clinician, which they were required to input when registering in the app. The NPI for each clinician indicates the state in which they gained their license to practice as a health care provider.

<u>Stimuli Design</u>

Our study included seven different clinical vignettes. The vignettes were selected based on two criteria: Each vignette *i*) had a well-established correct diagnostic response, and *ii*) used a well-known clinical situation that has been found to elicit diagnostic error. The clinical scenarios used in this study included acute cardiac events (4), geriatric care and decline in activities of daily living (5), lower back pain (6), and diabetes-related cardiovascular illness (7). See *Clinical Vignettes* in the supplementary materials for all the vignettes used in the experiment.

Every vignette was displayed in the app with an image of the patient followed by a case description (Figure S3). Each round, clinicians were given a question concerning the medical status of a patient and were asked to enter a diagnostic estimate in the "provide estimate" field. In some cases, clinicians were asked to estimate the probability (from 0 – 100) that participants had a particular condition, and in other cases, they were asked to estimate the participant's medical risk of a future adverse health event (from 0 – 100). The "treatment option" field provided a drop-down menu from which clinicians selected a treatment response for the patient in the vignette. The case description for each vignette was designed in consultation with clinicians to represent the type of questions that clinicians regularly face in board exams, where each question has a correct answer for both the probability of the specific condition and the correct course of treatment. Clinicians

were rewarded money based on the accuracy of their diagnostic estimate, and correctness of their treatment recommendation, where the maximum payment for the correct answer was $40 for each vignette. 75% of a clinician's payment was based on an accurate estimate ($30 if the answer was within 1 percentage point of the correct answer, $22 if within 10 percentage point, $15 if within 20 percentage point, $0 otherwise), and 25% was based on the correctness of their treatment recommendation ($10 if the correct treatment was selected, $0 otherwise). Participants were informed only that they would be rewarded based on the correctness of their responses up to $40 per trial, and were not given information about the specific payoff structure prior to completing the study.

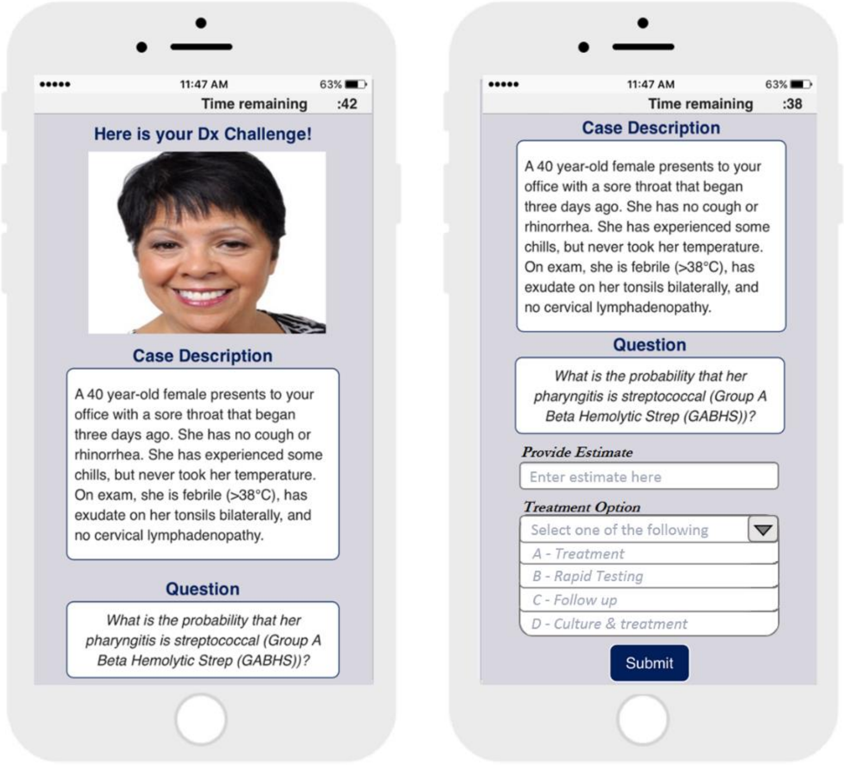

Figure S3. Image of the app and a sample vignette.

Participant Experience during the Experiment

Once participants registered using the DxChallenge app, they were informed that they would receive push notifications on their phone when the next diagnostic challenge was taking place (Figure S4). Clinicians clicked the push notification and entered the trial if they were available and interested in playing at the time the push notification appeared on their screen. When the number of clinicians needed to fill each condition was met, the clinicians who responded to the notification were randomized to conditions and the experimental trial began. If clinicians navigated away from the app either before or during the trial, they received additional push notifications to notify them to return to the app to complete the challenge. No clinician was allowed to participate in the same clinical case multiple times. Each trial was an independent test of a unique clinical case. Participating clinicians would undergo an independent randomization process for each trial.

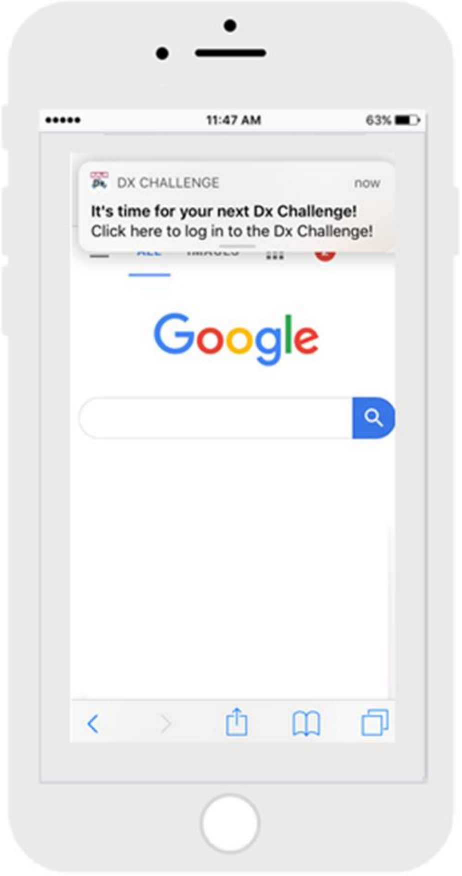

Figure S4. Displayed is a screenshot of the push notification clinicians received to invite them to participate in a diagnostic challenge.

All clinicians in the same trial were shown the same vignette. At round one, each clinician was asked to input a diagnostic estimate and a choice of treatment from a set of options in a dropdown menu. At round 2 and 3 in the control condition, clinicians were shown the same vignette and were asked to answer the same question on their own, with no change to the user experience. At round 2 and 3 in the network condition, clinicians were shown the average answer of the clinicians they were connected to in the social network structured through the DxChallenge app, and they were once again asked to provide a diagnostic estimate and to select a treatment option (Figure S5). The participant experience was identical between the control and the network condition, except for that participants in the network condition were exposed to the average estimate of the other clinicians they were connected to in the network. If at any point a player attempted to advance to the next round without inputting a diagnostic estimate or a treatment choice, a message appeared telling them that they had to input all required responses before advancing.

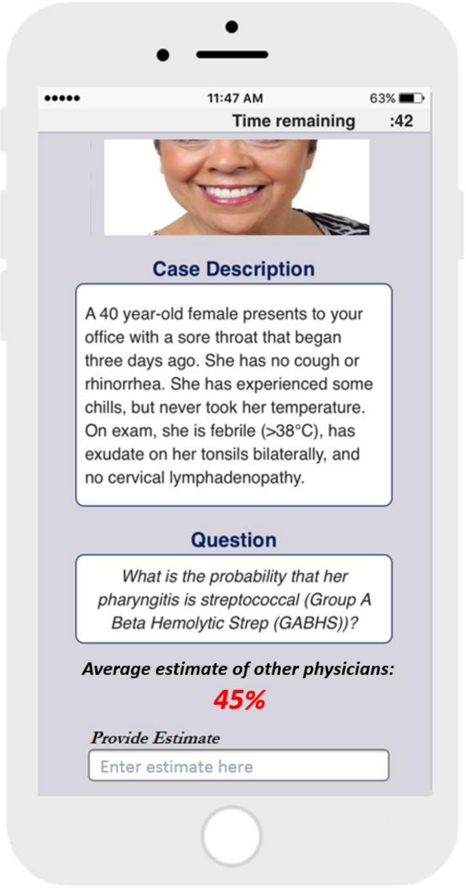

Figure S5. Displayed is a screenshot of the how the average estimate of clinician peers was presented during round 2 and round 3 in the network condition.

When each trial finished, clinicians were brought to a final payment page that displayed (i) the amount of money they won based on their accuracy, (ii) the correct answers to the questions in the vignette, and (iii) resources for learning more about the specific condition examined in the vignette

(Figure S6). All participants, regardless of condition, were provided with the same information and resources regarding the correct answer for a given vignette.

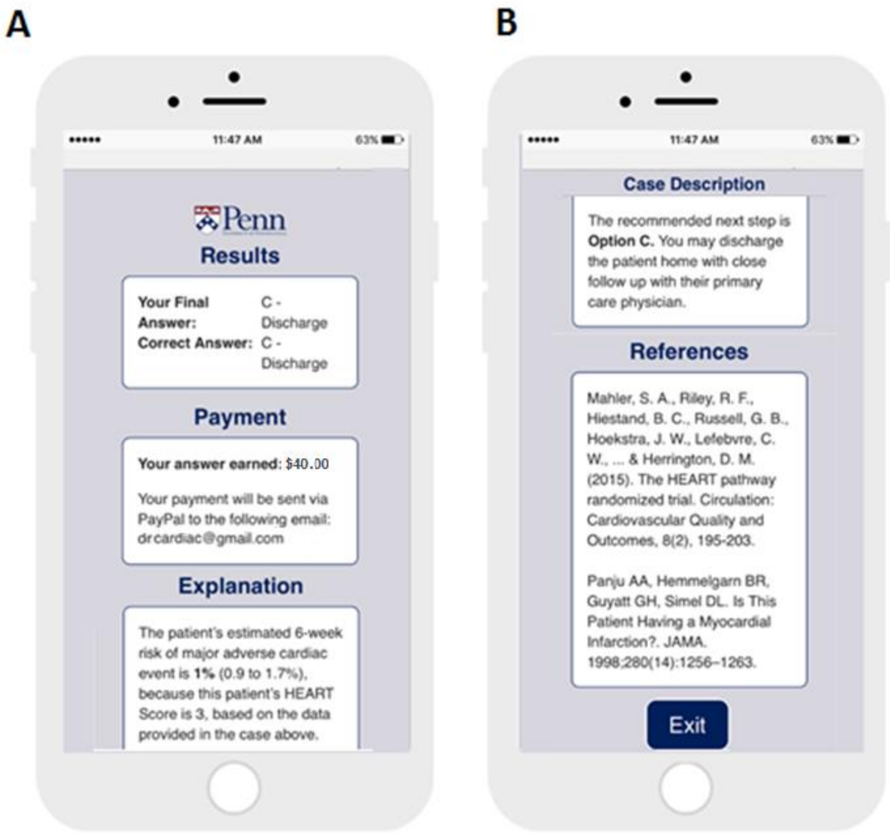

Figure S6. Displayed is a sample screenshot of the payout page that clinicians saw after each trial completed. On most devices, clinicians had to scroll down to view all the information provided. Panel A displays the initial results displayed on the payout page, and panel B displays the remaining information that could be viewed by scrolling down.

Clinical Vignettes

The use of case-based vignettes is standard for assessing quality of clinical decision-making, and is the basis for providing clinicians with board-certification (8-10). The case vignettes used in this study are general medicine cases that adult-treating generalists (such as internists, family physicians, or emergency physicians) would be expected to encounter during the course of clinical care. Even clinicians trained in other specialties are expected to have encountered similar clinical decisions and would be comfortable making an initial assessment for these patients.

Within this study, we were not able to assess the impact of our design on less typical cases, for instance with disparate difficulty levels, to determine the broader range of situations in which the rarity of the cases might affect the impact of our experimental intervention. Prior studies have shown that clinicians are more open to feedback on cases where they perceive more diagnostic uncertainty or lack confidence in the diagnosis, so we anticipate that this intervention would be more effective in clinical cases with greater uncertainty (8,9). Our findings on the

effects of structured network learning on the quality of clinician performance on the typical cases used in this study suggests that an important direction for future research is to explore a broader range of cases across narrower specialties to evaluate the application of these network effects in fields outside of generalist care.

Below are all the vignettes used in the experiment. Each vignette was displayed with a patient image that matches the age range and gender of the hypothetical patient in the vignette. The background image of all patients was edited out to form a uniform white background, allowing us to standardize contextual features of each image across all vignettes and trials.

The correct response for each vignette is based on available evidence-based research and guidelines for care. For vignettes 1, 2, 4, 5, 6, and 7, the correct response is identified by existing society guidelines for evidence-based care. In the case that no society guidelines exist (i.e., vignette 3), the correct response is determined by available evidence-based research (see below).

1. Diabetes-related Cardiovascular Illness Prevention

Case description: A 41 year-old male comes in for an annual visit. He has well-controlled Type 2 Diabetes on oral agents. He denies hypertension and is a nonsmoker. On exam, his blood pressure is 130/70. His total cholesterol is 150 and HDL 35.

Question (Diagnostic Estimate): Based on this assessment, what is his estimated lifetime risk of atherosclerotic cardiovascular disease?

Question (Treatment): Would you recommend a moderate intensity statin in this patient?

- A – Yes
- B – No

Response with Explanation: Yes, as the AHA/ACC guidelines state that a moderate-intensity statin therapy should be initiated or maintained for adults 40 to 75 years of age with diabetes mellitus. In this patient, the estimated life risk of atherosclerotic cardiovascular disease (ASCVD) is 50%, regardless of race/ethnicity.

References: see supplementary reference 11.

2. Adverse Cardiac Event

Case description: The 54 year-old patient pictured above, presents with 2 hours of burning chest pain to the emergency department. The pain began at rest, and it radiated to his back and left axilla. The patient reports no other complaints and reported being in good health otherwise. The past medical history was notable for a history of depression, a 40-pack/year history of cigarette smoking, and a parent who had a heart attack at age 49. On physical examination, the initial blood pressure was elevated at 192/100 mm Hg, but heart rate and the rest of the cardiopulmonary

examination were normal. Bilateral upper-extremity blood pressures were equal. You obtain an electrocardiogram (ECG) shown here.

A chest radiograph and routine blood work, including a troponin assay, were also normal. The patient received aspirin and sublingual nitroglycerin without symptom improvement. You give a "GI cocktail" (an oral antacid/anesthetic combination) and the patient reports symptom relief with normalization of the initial blood pressure.

Question (Diagnostic Estimate): What is the estimated 6-week risk of this patient having a major adverse cardiac event?

Question (Treatment): What is your recommended next step?

- A – Discharge patient home with follow up with primary care clinician
- B – Admit to observational unit for repeat EKG and cardiac biomarker testing

Response with explanation: Based on the data provided in the case above, this patient's Heart Score is 4, with a 6-week risk of Major Adverse Cardiac Event (MACE) of 13%. Therefore, option b, observation with repeat cardiac enzymes and EKG is recommended. Burning pain has not been associated with a reduced likelihood of ischemia and chest pain radiating to left axilla is associated with an increased likelihood of ischemia. The HEART (history, ECG, age, risk factors, and troponin) score is a composite risk-stratification tool that uses information readily available to the emergency physician at the point when a disposition and plan must be made. The HEART Score is supported by The American College of Emergency Physicians (ACEP) with Level 1a evidence (Evidence obtained from Meta Analysis of Randomized Trials.)

References: see supplementary references 12-16.

3. Geriatrics

Case description: A 75 year-old patient was admitted to the Medical Surgical floor after being diagnosed with a urinary tract infection and a contusion of the left hip secondary to a fall. Imaging of the hip, pelvic, and lower spine revealed no fractures. Past medical history is significant for hypertension, stable on an ace inhibitor, as well as hypothyroidism, stable on levothyroxine. On hospital day 3, you are called to evaluate the patient for discharge, who is now on oral antibiotics and non-narcotic analgesics for pain. The patient has been receiving physical therapy. The patient is now dependent in bathing (ADL) and house cleaning (IADL) due to hip pain.

Question (Diagnostic Estimate): What is the likelihood that this patient would experience a decline in ADL (Activities of Daily Living) function between hospital admission and discharge.

Question (Treatment): What would guide your discharge recommendations for this patient?

- A - Defer to case manager
- B - Defer to physical therapist
- C - Discharge to post-acute care
- D - Defer to patient preference

Response with Explanation: 16% of patients aged 75-79, as prior studies estimate, experience a decline in ADL function between their pre-hospital baseline and discharge. What should guide your recommendations? Option D. IADLs can be supplemented or adapted to allow people to remain in the community. Functional decline during hospitalization is common. Therefore, engaging physical therapy and occupational therapy early in care is essential. The six factors associated with the need for a post-acute care referral included patients who had: no or intermittent help available, major walking restrictions, less than excellent self-rated health, longer lengths of stay, higher depression scores or higher number of co-morbidities. This patient had some walking restrictions, no depression and few co-morbidities. Therefore, patient-centered care of older adults involves eliciting their goals and preferences to guide your recommendations. For more information, refer to Table 2 in this summary of evidence (Covinsky et al. 2011), and additional references below.

References: see supplementary references 17-22.

4. Acute MI

Case description: A 76-year-old patient with history of hypertension, diabetes, and advanced dementia was brought to the emergency department (ED) from a nursing facility with confusion and generalized weakness. Based on initial evaluation, the patient was diagnosed with a urinary tract infection and started on antibiotics in the ED. As part of this evaluation, the patient was found to have a mildly elevated troponin I level (0.10 µg/mL; normal is < 0.07 µg/mL). The patient's electrocardiogram (ECG) was unchanged from the baseline and showed no evidence of ischemia. The patient did not complain of chest pain or shortness of breath. You consult a cardiologist for evaluation of the elevated troponin level. The cardiologist started the patient on aspirin, clopidogrel, and heparin for treatment of possible non–ST-elevation myocardial infarction (NSTEMI). The following day, the patient remained confused. The patient's troponin level rose to 0.13 µg/mL and was 0.12 µg/mL on repeat. The ECG continued to show no evidence of myocardial ischemia.

Question (Diagnostic Estimate): What is the likelihood that this patient is having an acute MI?

Question (Treatment): What would be your recommended next steps?

- A - Discontinue treatment
- B - Continue current medication

Response with explanation: The pretest probability (i.e., likelihood) that a patient with confusion and weakness, without chest pain or dyspnea, with normal vital signs, and a non-ischemic ECG is having an acute MI is zero. In patients whose pretest probability of a disease is low, a positive test result for that disease is likely to be a false positive. Troponin testing should be reserved for patients with signs and symptoms consistent with myocardial ischemia.

References: see supplementary reference 23 and 24.

5. Back Pain

Case description: A 48-year-old patient is evaluated in clinic with a 3-day history of low back pain without leg pain. The patient has no previous history of cancer, no weight loss, anorexia, fevers, or night sweats and does not recall any specific work-related injury. Your physical examination reveals mild paralumbar tenderness with normal strength, sensation, and lower extremity reflexes. The patient has not worked for 3 days due to the back pain and rates the pain as 8 out of 10 with little improvement with over-the-counter acetaminophen. The past medical history is significant only for chronic depression.

Question (Diagnostic Estimate): What is the probability that this patient will require immediate imaging?

Question (Treatment): What would be your recommended next steps?

- A - Encourage to remain active
- B - Refer to therapy
- C - Order an MRI

Response with explanation: Only 5% of patients seen with these symptoms will require immediate imaging. Any patient with symptoms of spinal cord compression, progressive, and/or severe neurologic deficits should have immediate MRI for further evaluation and urgent specialist referral. Such symptoms and signs include new urinary retention, incontinence from bladder overflow, new fecal incontinence, saddle anesthesia, and significant motor deficits not localized to a single unilateral nerve root. Other patients who may require imaging on initial evaluation include those with a high suspicion for spinal infection, a current or recent history of cancer, major risk factors for cancer, major trauma to area, and those with suspected vertebral compression fracture. Encouraging patients to remain active is key to symptom resolution and 90% of patients with acute nonspecific low back pain will recover within two weeks. After 4 weeks, with persistent symptoms, referral to physical therapy is an option.

References: see supplementary reference 25.

6. Adverse Cardiovascular Event

A 62-year-old patient presents to the emergency department with sharp chest pain which occurred earlier that day while at a desk at work. The pain worsened with movement or taking a deep breath. The patient was otherwise healthy and not reporting any other complaints. The patient reports smoking a pack per day. The past medical history was otherwise unremarkable. Family history was notable for a sister who had a heart attack at age 67. The patient's body mass index (BMI) is 24. On physical examination, the patient's initial blood pressure was mildly elevated at 150/90 mm Hg, but the heart rate and the rest of the cardiopulmonary examination were normal. Bilateral upper-extremity blood pressures were equal. You obtain an electrocardiogram (ECG) which shows a non-specific repolarization disturbance. A chest radiograph and routine blood work, including a troponin assay, were normal.

Question (Diagnostic Estimate): What is the estimated 6-week risk of this patient having a major adverse cardiac event?

Question (Treatment): What would be your recommended next steps?

- A - Admit to obs. Unit
- B - Admit to hospital
- C – Discharge

Response with explanation: The patient's estimated 6-week risk of major adverse cardiac event is 1% (0.9 to 1.7%), because this patient's HEART Score is 3, based on the data provided in the case above. The recommended next step is Option C. You may discharge the patient home with close follow up with their primary care clinician.

The HEART (history, ECG, age, risk factors, and troponin) score is a composite risk-stratification tool that uses information readily available to the emergency physician at the point when a disposition and plan must be made. The HEART Score is supported by The American College of Emergency Physicians (ACEP) with Level 1a evidence (Evidence obtained from Meta Analysis of Randomized Trials.)

References: see supplementary references 14–16, 26 and 27.

7. Adverse Cardiac Event

A 67-year-old patient, with a history of hypertension, well-controlled on one medication, presents to the emergency room after noticing chest pain while climbing up one flight of stairs this morning, but felt a bit better after sitting down. The patient's physical exam including vitals are unremarkable. The patient has a body mass index (BMI) of 32, and reports being a former smoker, having quit one month prior to this visit. The patient's family history is notable for a father who had a heart attack at age 62. The electrocardiogram (ECG) reveals LVH, the chest radiograph reveals no abnormalities, and the initial troponin assay is within normal limits.

Question (Diagnostic Estimate): What is the estimated 6-week risk of this patient having a major adverse cardiac event?

Question (Treatment): What would be your recommended next steps?

- A – Discharge
- B - Admit for Evaluation
- C - Admit for Obs. Unit

Response with explanation: The patient's estimated 6-week risk of this patient having a major adverse cardiac event is 57% (50 to 65%), because this patient's HEART Score is 7, based on the data provided in the case above. The recommended next step is Option B. This patient should be admitted to the hospital inpatient unit for further cardiac evaluation.

The HEART (history, ECG, age, risk factors, and troponin) score is a composite risk-stratification tool that uses information readily available to the emergency physician at the point when a disposition and plan must be made. The HEART Score is supported by The American College of Emergency Physicians (ACEP) with Level 1a evidence (Evidence obtained from Meta Analysis of Randomized Trials.)

References: see supplementary references 14–16, 26 and 27.

Statistical Information

Each trial of this study is independent from one another, and each condition within each trial is independent from one another. To compare the diagnostic accuracy of participants across experimental conditions, we first compute the average diagnostic accuracy of participants in each experimental condition within each trial. This approach produces an independent, group-level measure of accuracy for each trial in each experimental condition. To compare experimental conditions across trials, we use the nonparametric Wilcoxon test. All comparisons using the Wilcoxon test are two-tailed to not only test for the hypothesis that social learning increases accuracy, relative to controls, but also for the possibility that it decreases accuracy. We used this methodology to compare conditions in terms of their (i) initial and (ii) final diagnostic accuracy, as well as average change in diagnostic accuracy for individuals in each condition (measured by subtracting their initial round one diagnostic accuracy from their final round three accuracy). We additionally examine the correlation between clinicians' initial diagnostic accuracy and the extent to which they revised their diagnostic estimates, from the first to final round.

Supplementary Analyses

Fig. S7 elaborates the results reporting in Figure 2 in the main text, showing the correlation between revision magnitude and initial error at the individual level.

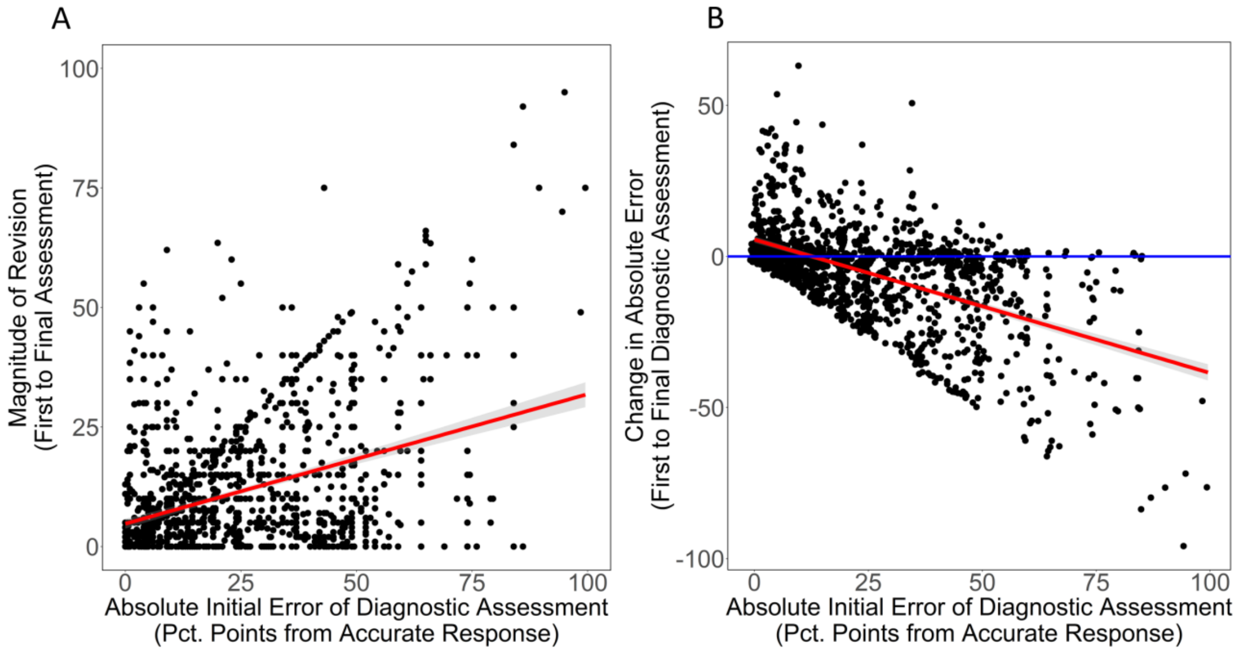

Figure S7. The correlation between clinicians' initial accuracy and their use of social information to improve their assessments. Panel (a) shows clinicians' propensity to revise their diagnostic assessments (from first to final assessment) based on social influence as a function of their initial error. Panel (b) shows the extent to which clinicians' revisions based on social influence (from first to final assessment) led to improvements in their final diagnostic assessment, as a function of their initial error. The red trend line reflects a standard OLS correlation. The blue horizontal line in panel (b) marks the axis indicating no change in accuracy. Values above this line indicate an increase in error, and values below this line indicate a reduction in error. Error bands show 95% confidence intervals.

Figure S7A shows, as expected, that there is a significant positive correlation between the absolute initial error of clinicians' diagnostic assessment and the extent to which clinicians revised their diagnostic assessment ($p < 0.001$, $r = 0.4$, CI=[0.35,0.43]); specifically, less accurate clinicians made greater revisions to their responses while more accurate clinicians made smaller revisions, giving greater *de facto* influence in the social network to more accurate clinicians. The positive revision coefficient translated into significant improvements in the overall accuracy of clinicians' diagnostic assessments. Figure S7B shows that clinicians with the highest initial error were likely to improve their accuracy substantially as a function of social learning ($p < 0.001$, $r = -0.54$, CI=[-0.57,-0.5]).

Figure S8 compares the average performance across conditions for each of the 7 cases in this study. Consistent with the findings in the main text, trial level analyses (i.e., treating each network, or control group, as a single observation, rather than treating each individual subject as a single observation) show that networks are significantly more likely to improve across all vignettes (p=0.005, Wilcoxon Rank Test, n=84).

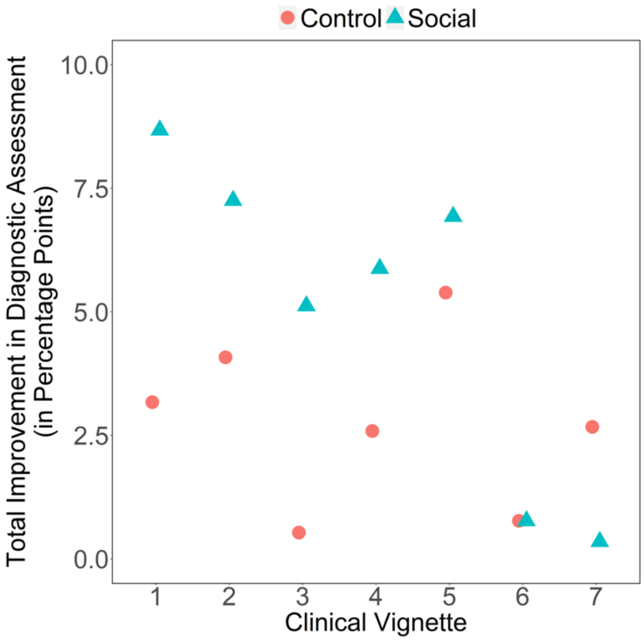

Figure S8. The average improvement (from the initial to the final round) of clinicians' diagnostic assessments, differentiated by clinical vignette. Blue triangles show the average performance of network trials, and red circles show the average performance of control trials.

Additionally, we also ran an OLS model that predicted the average individual improvement in diagnostic assessment within each trial as a function of the experimental condition (control vs. network), while also controlling for each vignette. Consistent with the trial level analyses, we find that clinicians in the network condition were significantly more likely to improve in their diagnostic assessment across all vignettes ($\beta = 2.25$, SE=0.78, t=2.87, p=0.005).

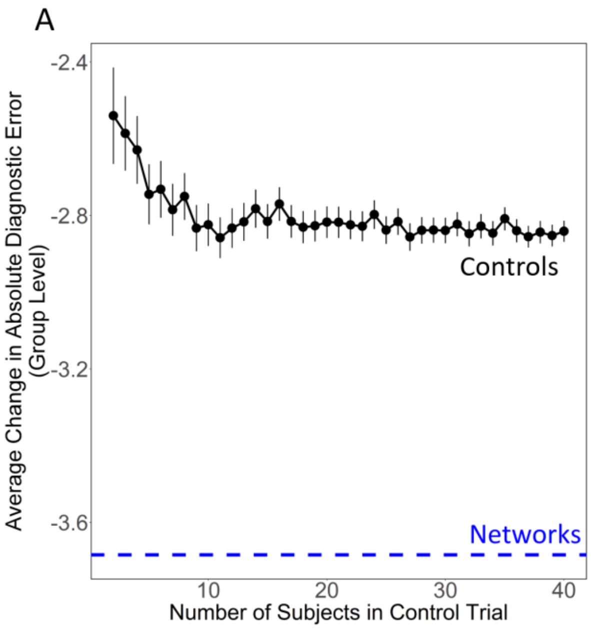

Figure S9. Comparing the performance of clinicians in the control and network condition across a wide range of possible group sizes for the control condition. Data are generated using a bootstrapping approach, where for each vignette, 1,000 unique groups of a particular size (indicated by the horizontal axis) are generated by sampling from all clinicians with replacement. This plot displays the absolute change of the error of the mean group assessment. The dashed blue line indicates the empirical performance achieved by applying the same metric to clinicians in the network condition. Error bars show 95% confidence intervals.

Figure S9 compares the average change in absolute diagnostic error observed in the network condition to the same outcome measured in control groups of varying size. Data are generated using a bootstrapping approach, where for each vignette, 1,000 unique groups of a particular size (indicated by the horizontal axis) are generated by sampling from all clinicians with replacement. Figure S9 shows that networks produce more accurate collective judgments than control groups, regardless of the size of control group (p<0.0001, Wilcoxon Signed-Rank Test).

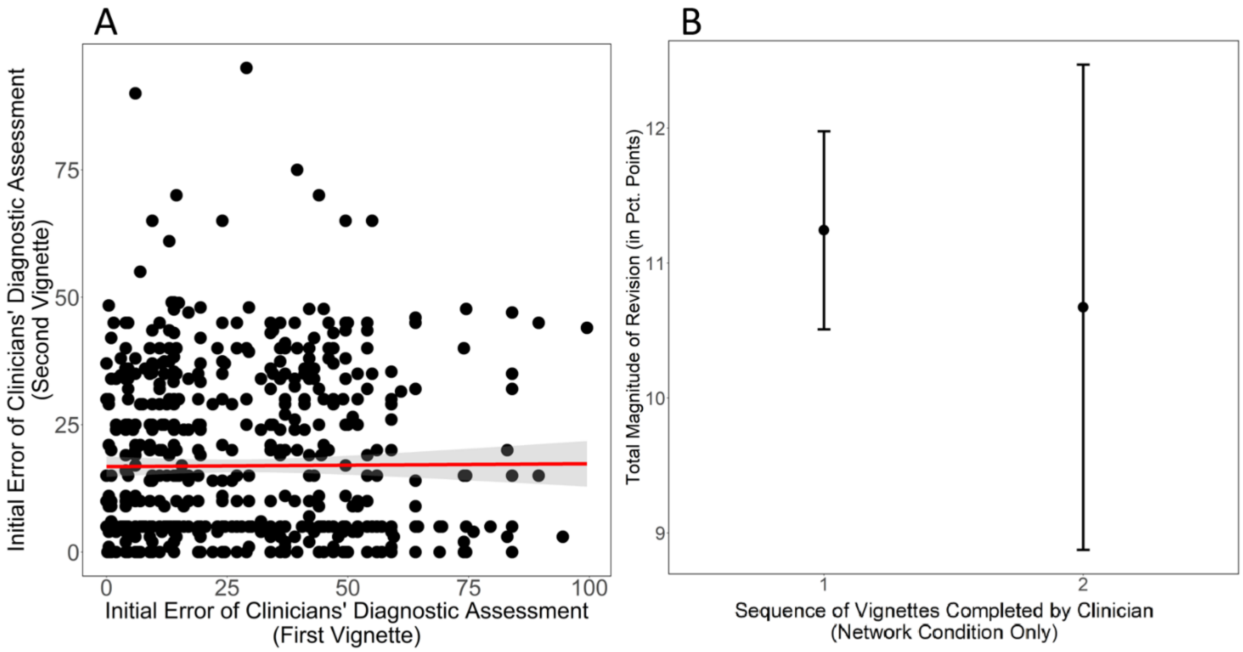

Figure S10. (A) Comparing clinicians' initial error in their diagnostic assessment across vignettes, for those 30% of participating clinicians who completed two separate vignettes. Error band displays 95% confidence intervals. (B) For those clinicians who completed two vignettes in the network condition, we display their average magnitude of revision (from the first to final assessment) in the first and second vignette they completed.

Lastly, we evaluate the 30% of clinicians in our experiment who completed two unique vignettes. First, panel A of Fig. S10 shows that there is no significant correlation between the initial error in clinicians' diagnostic assessments across the first and second vignette they completed. Consistent with prior work (1), this indicates that clinicians who are accurate in one canonical setting are not consistently accurate in another. Moreover, this helps address the concern about whether clinicians who completed two vignettes may have gained task-specific expertise that could have helped them perform better at baseline in their second vignette. Instead, we find that participating in a prior vignette provides no advantage in terms of clinicians' baseline accuracy on the subsequent vignette. This is corroborated by panel B of figure S10, which examines only those clinicians who completed two vignettes in the network condition, with the finding that there is no significant difference in the extent to which these clinicians used social information and updated their diagnostic estimates across vignettes. This rules out the concern that clinicians who participate in multiple vignettes in the network condition may have learned to follow the social information as a way of obtaining more money in the task. Instead, we find that participating in a prior vignette in the network condition does not make a clinician more likely to leverage social information when revising their opinion in the second vignette they complete in the network condition.

Implications for Practical Application

The practical value of our findings, and their implications for real-world implementation are significantly increased by the growing adoption of electronic consultations (e-consult) systems. E-consults provide an infrastructure similar to the one employed in our study, which can allow this type of network intervention to be incorporated into existing workflows (e.g., in ambulatory care settings). Currently with e-consults, a primary care provider (PCP) may have a clinical question for a specialty consultant that does not require an immediate response; for instance, requiring follow-up within 5-7 days. In health systems that have adopted electronic consultations, clinicians send a formal query through e-consult to the specialist. The specialist will then respond with advice or thoughts within a window of several days. Expanding on this existing practice, we envision that with slight changes in technology and current infrastructure, the formal query could be sent to multiple specialists who are members of a clinical review network. Each member of the network would participate in an iterative process of providing recommendations, then being updated on other network members' responses, and then revising their recommendations at their own convenience within a 24hr window. After two iterations, the collective recommendation would then be returned back to the PCP. This network approach to consultation is made increasingly realistic by the fact that existing networks of specialists connected virtually (such as those in organizations like RubiconMD (28)) have been growing rapidly within the last few years.

Table S1: Summary statistics for outcome measures by initial assessment error quartiles, by vignette, and by experiment conditions, averaged across trials.

Experiment Condition	Initial Error Quartile	Vignette	Initial Assessment Accuracy	Final Assessment Accuracy	Change in Accuracy	Initial Rate of Correct Treatment	Final Rate of Correct Treatment	Change in Treatment
Control	Q1 (error)	1	0.98	0.97	-0.01	0.71	0.74	0.03
Control	Q2 (error)	1	0.90	0.92	0.02	0.58	0.52	-0.06
Control	Q3 (error)	1	0.66	0.70	0.04	0.45	0.64	0.19
Control	Q4 (error)	1	0.43	0.60	0.17	0.75	0.44	-0.31
Network	Q1 (error)	1	0.98	0.96	-0.02	0.76	0.65	-0.11
Network	Q2 (error)	1	0.90	0.93	0.03	0.60	0.57	-0.03
Network	Q3 (error)	1	0.70	0.88	0.18	0.74	0.64	-0.10
Network	Q4 (error)	1	0.46	0.76	0.30	0.41	0.50	0.09
Control	Q1 (error)	2	0.99	0.85	-0.14	0.60	0.60	0.00
Control	Q2 (error)	2	0.89	0.92	0.03	0.42	0.50	0.08
Control	Q3 (error)	2	0.70	0.77	0.07	0.40	0.50	0.10
Control	Q4 (error)	2	0.57	0.60	0.03	0.40	0.40	0.00
Network	Q1 (error)	2	0.98	0.89	-0.09	0.59	0.71	0.12
Network	Q2 (error)	2	0.89	0.86	-0.03	0.39	0.58	0.19
Network	Q3 (error)	2	0.71	0.80	0.09	0.34	0.49	0.15
Network	Q4 (error)	2	0.56	0.71	0.15	0.36	0.62	0.26
Control	Q1 (error)	3	0.97	0.96	-0.01	0.94	0.92	-0.02
Control	Q2 (error)	3	0.86	0.92	0.06	0.88	0.75	-0.13

Control	Q3 (error)	3	0.73	0.74	0.01	0.50	0.62	0.12
Control	Q4 (error)	3	0.40	0.65	0.25	0.43	0.57	0.14
Network	Q1 (error)	3	0.96	0.97	0.01	0.89	0.97	0.08
Network	Q2 (error)	3	0.87	0.96	0.09	0.79	0.96	0.17
Network	Q3 (error)	3	0.77	0.94	0.17	0.79	0.74	-0.05
Network	Q4 (error)	3	0.33	0.96	0.63	0.36	0.91	0.55
Control	Q1 (error)	4	0.98	0.97	-0.01	0.67	0.72	0.05
Control	Q2 (error)	4	0.88	0.90	0.02	0.23	0.33	0.10
Control	Q3 (error)	4	0.74	0.81	0.07	0.21	0.21	0.00
Control	Q4 (error)	4	0.47	0.63	0.16	0.09	0.27	0.18
Network	Q1 (error)	4	0.98	0.96	-0.02	0.61	0.61	0.00
Network	Q2 (error)	4	0.88	0.91	0.03	0.24	0.34	0.10
Network	Q3 (error)	4	0.74	0.84	0.10	0.12	0.21	0.09
Network	Q4 (error)	4	0.45	0.79	0.34	0.05	0.19	0.14
Control	Q1 (error)	5	0.97	0.96	-0.01	0.89	0.95	0.06
Control	Q2 (error)	5	0.89	0.89	0.00	0.62	0.78	0.16
Control	Q3 (error)	5	0.75	0.77	0.02	0.56	0.44	-0.12
Control	Q4 (error)	5	0.43	0.64	0.21	0.15	0.38	0.23
Network	Q1 (error)	5	0.97	0.93	-0.04	0.89	0.88	-0.01
Network	Q2 (error)	5	0.89	0.88	-0.01	0.59	0.56	-0.03
Network	Q3 (error)	5	0.77	0.85	0.08	0.46	0.61	0.15
Network	Q4 (error)	5	0.40	0.70	0.30	0.27	0.47	0.20
Control	Q1 (error)	6	0.98	0.98	0.00	0.67	0.67	0.00
Control	Q2 (error)	6	0.89	0.75	-0.14	0.33	0.22	-0.11
Control	Q3 (error)	6	0.69	0.68	-0.01	0.27	0.30	0.03
Control	Q4 (error)	6	0.53	0.56	0.03	0.18	0.28	0.10
Network	Q1 (error)	6	0.97	0.81	-0.16	0.38	0.23	-0.15
Network	Q2 (error)	6	0.93	0.74	-0.19	0.29	0.24	-0.05
Network	Q3 (error)	6	0.69	0.68	-0.01	0.24	0.37	0.13
Network	Q4 (error)	6	0.53	0.59	0.06	0.13	0.22	0.09
Control	Q1 (error)	7	0.96	0.82	-0.14	0.31	0.38	0.07
Control	Q2 (error)	7	0.90	0.88	-0.02	0.22	0.43	0.21
Control	Q3 (error)	7	0.71	0.71	0.00	0.17	0.17	0.00
Control	Q4 (error)	7	0.39	0.47	0.08	0.21	0.25	0.04
Network	Q1 (error)	7	0.97	0.74	-0.23	0.08	0.15	0.07
Network	Q2 (error)	7	0.89	0.75	-0.14	0.26	0.28	0.02
Network	Q3 (error)	7	0.71	0.70	-0.01	0.15	0.29	0.14
Network	Q4 (error)	7	0.37	0.50	0.13	0.22	0.28	0.06

Table S2: Summary statistics for outcome measures by initial assessment error quartiles and by experiment conditions, aggregated at the trial level and averaged across trials.

Experiment Condition	Initial Error Quartile	Initial Assessment Accuracy	Final Assessment Accuracy	Change in Accuracy	Initial Rate of Correct Treatment	Final Rate of Correct Treatment	Change in Treatment
Control	Q1 (error)	0.97	0.94	-0.04	0.71	0.72	0.02
Control	Q2 (error)	0.89	0.88	-0.00	0.45	0.51	0.05
Control	Q3 (error)	0.71	0.74	0.03	0.36	0.38	0.03
Control	Q4 (error)	0.45	0.58	0.13	0.33	0.37	0.03
Network	Q1 (error)	0.97	0.88	-0.09	0.63	0.61	-0.01
Network	Q2 (error)	0.89	0.86	-0.03	0.46	0.52	0.06
Network	Q3 (error)	0.73	0.81	0.08	0.41	0.48	0.06
Network	Q4 (error)	0.44	0.71	0.28	0.24	0.43	0.19

Data Availability

The complete dataset is publicly available for download from the following URL: https://github.com/drguilbe/CIdiagnosis

References

1. J Becker, D Brackbill, D Centola, Network dynamics of social influence in the wisdom of crowds. *Proc Natl Acad Sci USA* **114**, E5070–E5076 (2017).
2. D Guilbeault, J Becker, D Centola, Social learning and partisan bias in the interpretation of climate trends. *Proc Natl Acad Sci USA* **115**, 9714–9719 (2018).
3. D Guilbeault, D Centola, Networked collective intelligence improves dissemination of scientific information regarding smoking risks. *PLoS One* **15**, e0227813 (2020).
4. JH Pope, *et al.*, Missed diagnoses of acute cardiac ischemia in the emergency department. *N Engl J Med* **342**, 1163–1170 (2000).
5. KE Covinsky, *et al.*, Loss of independence in activities of daily living in older adults hospitalized with medical illnesses: Increased vulnerability with age. *J Am Geriatr Soc* **51**, 451–458 (2003).
6. R Chou, *et al.*, Diagnosis and treatment of low back pain: A joint clinical practice guideline from the American College of Physicians and the American Pain Society. *Ann Intern Med* **147**, 478 (2007).
7. CM Gamboa, LD Colantonio, TM Brown, AP Carson, MM Safford, Race-sex differences in statin use and low-density lipoprotein cholesterol control among people with diabetes mellitus in the reasons for geographic and racial differences in stroke study. *J Am Heart Assoc* **6**, e004264 (2017).

8. AND Meyer, VL Payne, DW Meeks, R Rao, H Singh, Physicians' diagnostic accuracy, confidence, and resource requests: A vignette study. *JAMA Intern Med* **173**, 1952 (2013).
9. V Fontil, *et al.*, Testing and improving the acceptability of a web-based platform for collective intelligence to improve diagnostic accuracy in primary care clinics. *JAMIA Open* **2**, 40–48 (2019).
10. EC Khoong, *et al.*, Impact of digitally acquired peer diagnostic input on diagnostic confidence in outpatient cases: A pragmatic randomized trial. *J Am Med Inform Assoc* **28**, 632–637 (2021).
11. DC Goff, *et al.*, 2013 ACC/AHA guideline on the assessment of cardiovascular risk: A report of the American College of Cardiology/American Heart Association Task Force on Practice Guidelines. *Circulation* **129,** S49-73 (2014).
12. JH Pope, *et al.*, Missed diagnoses of acute cardiac ischemia in the emergency department. *N Engl J Med* **342**, 1163–1170 (2000).
13. TD Sequist, Missed opportunities in the primary care management of early acute ischemic heart disease. *Arch Intern Med* **166**, 2237 (2006).
14. SM Fernando, *et al.*, Prognostic accuracy of the HEART score for prediction of major adverse cardiac events in patients presenting with chest pain: A systematic review and meta-analysis. *Acad Emerg Med* **26**, 140–151 (2019).
15. SM Green, DL Schriger, A methodological appraisal of the HEART score and its variants. *Ann Emerg Med* **78**, 253–266 (2021).
16. J Laureano-Phillips, *et al.*, HEART score risk stratification of low-risk chest pain patients in the emergency department: A systematic review and meta-analysis. *Ann Emerg Med* **74**, 187–203 (2019).
17. KE Covinsky, E Pierluissi, CB Johnston, Hospitalization-associated disability: "She was probably able to ambulate, but I'm not sure." *JAMA* **306** (2011).
18. KE Covinsky, *et al.*, Loss of independence in activities of daily living in older adults hospitalized with medical illnesses: Increased vulnerability with age. *J Am Geriatr Soc* **51**, 451–458 (2003).
19. KH Bowles, *et al.*, Post-acute referral decisions made by multidisciplinary experts compared to hospital clinicians and the patients' 12-week outcomes. *Med Care* **46**, 158–166 (2008).
20. KM Mehta, *et al.*, A clinical index to stratify hospitalized older adults according to risk for new-onset disability. *J Am Geriatr Soc* **59**, 1206–1216 (2011).
21. SK Inouye, *et al.*, A predictive index for functional decline in hospitalized elderly medical patients. *J Gen Intern Med* **8**, 645–652 (1993).
22. MA Sager, *et al.*, Hospital admission risk profile (HARP): identifying older patients at risk for functional decline following acute medical illness and hospitalization. *J Am Geriatr Soc* **44**, 251–257 (1996).
23. AK Manrai, G Bhatia, J Strymish, IS Kohane, SH Jain, Medicine's uncomfortable relationship with math: Calculating positive predictive value. *JAMA Intern Med* **174**, 991 (2014).
24. K Thygesen, *et al.*, Third universal definition of myocardial infarction. *Circulation* **126**, 2020–2035 (2012).
25. R Chou, *et al.*, Diagnosis and treatment of low back pain: A joint clinical practice guideline from the American College of Physicians and the American Pain Society. *Ann Intern Med* **147**, 478 (2007).

26. SA Mahler, *et al.*, The HEART pathway randomized trial: Identifying emergency department patients with acute chest pain for early discharge. *Circ Cardiovasc Qual Outcomes*. **8**, 195–203 (2015).
27. AA Panju, Is this patient having a myocardial infarction? *JAMA* **280**, 1256 (1998).
28. RubiconMD. (2023) Available at https://www.rubiconmd.com/. Accessed April 22, 2023.
